# The role of Intravascular Ultrasound in the management of spontaneous coronary artery dissection

**DOI:** 10.1186/1476-7120-6-24

**Published:** 2008-05-31

**Authors:** Jayanth R Arnold, Nick EJ West, William J van Gaal, Theodoros D Karamitsos, Adrian P Banning

**Affiliations:** 1 Department of Cardiology, John Radcliffe Hospital, Oxford, UK; 2 Department of Cardiology, Papworth Hospital, Papworth Everard, Cambridge, UK.

## Abstract

Primary or spontaneous coronary artery dissection (SCAD) is an unusual but increasingly recognized cause of acute myocardial ischemia and sudden cardiac death. Typically, SCAD presents in younger patients without conventional risk factors for coronary artery disease. It occurs more commonly in women than in men, and frequently during pregnancy or the postpartum period. Its pathophysiology is poorly understood, and there is considerable controversy regarding the optimal management of patients with SCAD-related myocardial ischemia. Therapeutic approaches include conservative medical therapy, coronary artery bypass graft surgery and percutaneous coronary intervention (PCI). We present four cases of SCAD to illustrate specific aspects of the presentation and management of this condition, with particular reference to the importance of intravascular ultrasound (IVUS) to aid diagnosis and guide subsequent PCI.

## Background

Spontaneous coronary artery dissection (SCAD) is an unusual cause of acute coronary syndromes and sudden cardiac death. The pathophysiology of SCAD remains uncertain, and considerable controversy surrounds the optimal management of patients with SCAD-related myocardial ischemia. Urgent coronary angiography is indicated if SCAD is suspected, and the diagnosis can be confirmed by intravascular ultrasound (IVUS). Percutaneous coronary intervention (PCI), coronary artery bypass grafting (CABG) and medical management have been described as management strategies. We present four cases of SCAD which were diagnosed and managed at a single centre, and we illustrate specific aspects of the presentation and management of this important condition, with particular focus on the importance of IVUS to aid diagnosis and guide subsequent PCI.

## Case 1

**Figure 1:**
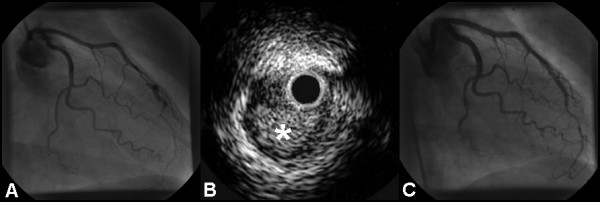
**(A) RAO caudal projection showing dissection of the LAD in the mid-vessel.(B) **IVUS examination showing hematoma (asterisk) compressing the true lumen. **(C) **RAO caudal view of the LAD demonstrating a favourable angiographic appearance following stent deployment.

A 54-year-old postmenopausal woman with no cardiovascular risk factors presented with a one week history of chest pain on exertion and at rest. The ECG revealed anterior T-wave inversion and the troponin I was elevated (0.5 ng/ml; local reference limit, 0.1 ng/ml). Despite treatment with aspirin, low-molecular weight heparin and beta-blockade, the patient remained symptomatic. Coronary angiography showed extensive dissection of the mid-vessel of the LAD (figure 1A, see additional file 1). In view of the long diseased segment and small distal vessel, percutaneous intervention was not undertaken; medical management was recommended, with beta-blockade and anti-platelet therapy.

However, the patient continued to experience exertional angina and an exercise stress test was positive at a low workload. Repeat angiography at three months showed persistent dissection. IVUS examination showed near-circumferential hematoma extending deep into the media (figure 1B). No atheroma was visualised. Two 3.0 × 18 mm Cypher™ stents (Cordis Corp., Miami, Florida) were deployed over the dissection with overlap. Repeat IVUS demonstrated incomplete stent deployment, and post-dilatation with a noncompliant balloon (3.0 × 15 mm) was undertaken, resulting in a favourable angiographic result (figure 1C, see additional file 2). IVUS confirmed complete sealing of the dissection, with full stent apposition. The patient was discharged on long-term aspirin and 6 months of clopidogrel. Follow up angiography two years later demonstrated an excellent result, and the patient remained symptom-free.

## Case 2

**Figure 2:**
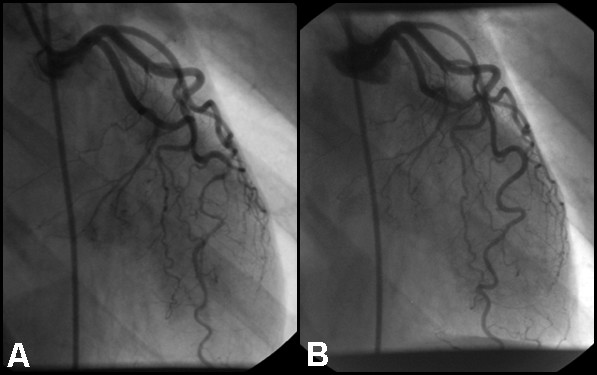
**(A)RAO caudal projection showing dissection of the LAD in the distal vessel at baseline, and(B) resolution of LAD changes at six month follow up.**

A 53 year old lady with no cardiovascular risk factors presented with left-sided chest pain and dyspnoea. The ECG showed infero-lateral T-wave inversion and the troponin I was elevated (18.5 ng/ml). Medical management was instituted, with resolution of symptoms. Coronary angiography revealed a tortuous LAD with angiographic appearances suggestive of spontaneous dissection but no limitation of antegrade flow (figure 2A). No intervention was undertaken owing to the tortuosity of the vessel and the absence of ongoing symptoms.

Six months later the patient underwent repeat angiography which demonstrated resolution of the changes in the LAD (figure 2B).

## Case 3

**Figure 3:**
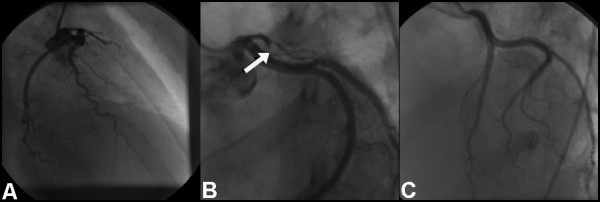
**(A) RAO caudal projection showing proximal occlusion of the LAD.(B) **LAO cranial projection demonstrating LAD occlusion with the tram-track appearance of dissection. **(C) **LAO cranial view showing a favourable angiographic appearance following stent deployment.

A 36-year old lady with no cardiac risk factors experienced acute onset of chest pain and brief syncope following an argument. The ECG revealed anterior ST elevation and trans-thoracic Echocardiography confirmed antero-apical hypokinesia. Thrombolysis with tissue plasminogen activator was given. The patient's condition worsened so she was transferred to the local tertiary centre where diagnostic angiography revealed proximal occlusion of the LAD due to coronary dissection (figure 3A, 3B). A guidewire was navigated with difficulty and two stents were deployed (3.0/32 mm Express and 3.5/22 mm Biodivysio) with restoration of blood flow (figure 3C). An excellent angiographic result was achieved. At follow up three years later, the patient remained symptom-free.

## Case 4

**Figure 4:**
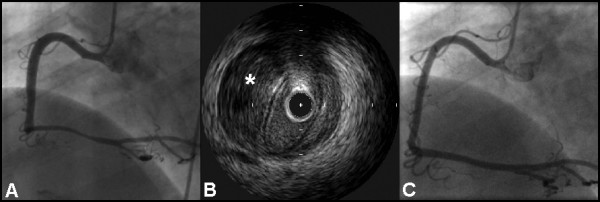
**(A) LAO view showing long stenosis of the RCA, and(B) corresponding IVUS examination showing hematoma (asterisk) compressing the true lumen.(C) LAO projection showing a favourable angiographic appearance at follow up.**

A 59-year old lady presented with central chest pain. She had a history of a non-ST-elevation myocardial infarction four years previously; she was an ex-smoker and had hypertension and hypercholesterolemia. The initial ECG revealed lateral T-wave inversion and the troponin I was elevated (5.7 ng/ml). Treatment with aspirin, low-molecular weight heparin and beta-blocker was commenced. Despite initial relief of symptoms, the patient developed further chest pain three days later. The ECG showed lateral ST elevation. Thrombolysis with reteplase was given. However, there was inadequate resolution of the ST segments and the patient's symptoms worsened. She was therefore transferred to the local tertiary centre. Angiography revealed a long tubular stenosis in a dominant right coronary artery, from the crux and throughout the proximal and mid-PDA (figure 4A, see additional file 3). IVUS was undertaken and this confirmed extensive intramural hematoma caused by coronary dissection (figure 4B, see additional file 4). In view of the length of the diseased segment and small distal vessel, percutaneous intervention was not undertaken, and medical management was continued. At follow up four months later, the patient remained symptom-free and repeat angiography demonstrated healing of the previously dissected right coronary artery (figure 4C, see additional file 5).

## Discussion

Since its first description [1], SCAD has been reported repeatedly in the literature, but rarely in large enough series to draw clear conclusions regarding its aetiology, pathophysiology or optimal management. Previous studies show that SCAD is commonest in the fifth decade, with a striking female predominance, particularly occurring in the peripartum period [2-6]. Furthermore, it has been described in association with exercise [7], cocaine abuse [8] and oral contraceptive use [9] in previously healthy individuals, and also in a variety of connective tissue and vasculitic disorders [10,11].

Short term mortality following SCAD is higher than with acute coronary syndromes and ST-elevation myocardial infarction. In a review of 222 cases of peripartum SCAD, short-term mortality was 43%, but the survival rate was 95% if the initial phase was survived[12] Prognosis is poorer in cases with left main stem, LAD and multivessel involvement, which typically result in extensive infarction or death. Generally, prognosis is worse in women and worse in non-peripartum women[4].

The principal abnormality observed in SCAD is the development of hematoma in the outer third of the vessel media, causing luminal encroachment [13]. When present, an intimal tear may decompress hematoma in the false lumen and protect against vessel occlusion.

Considerable controversy surrounds the aetiology of SCAD. Although atherosclerotic plaque rupture has been suggested as a possible mechanism, this seems unlikely given the frequent absence of atherosclerotic risk factors and the relatively early age of onset. Also postulated is spontaneous rupture of vasa vasorum in the vessel media, occurring as a result of vascular shear stress or abnormal connective tissue structure. The eosinophilic infiltrates observed in some cases may damage collagen and lead to cystic medial necrosis [11] and progesterone-induced microstructural changes may be important in peri-partum and contraceptive-associated SCAD [3,4]. However, these changes are not universally observed, lending weight to the hypothesis that several aetiologies may end with the final common pathway of SCAD [14]. There are also numerous reports of SCAD being precipitated by acute changes in intra-thoracic pressure (e.g. retching, sneezing, vigorous exercise, sexual intercourse [7,15,16]). However, most cases of peripartum SCAD do not present during delivery, but at an average time-delay of nearly one month [17].

Although SCAD usually presents with symptoms of myocardial ischemia, it may also present with cardiogenic shock or sudden death. Any presentation of an acute coronary syndrome in a young patient without conventional risk factors or in a woman in the peripartum period should raise suspicion of SCAD. Diagnosis of SCAD is generally made by coronary angiography, with the appearance of a radiolucent intimal flap or slow clearance of contrast from the false lumen. In consecutive coronary angiography series, the incidence of SCAD has been reported from 0.1% to 1.1% [2,3]. However, SCAD may elude diagnosis even with angiography: if an intimal tear is absent, the medial hematoma may appear as a narrowed or occluded vessel with coronary angiography.

Therefore, using angiography alone, the true incidence of SCAD is likely to be underestimated. However, a definitive diagnosis may be provided by using IVUS, which will distinguish atherosclerotic stenosis from intimal hematoma (see table 1). One large IVUS database (comprising over 15,000 patients) identified five cases of unsuspected SCAD in a pre-PCI population without angiographic features to suggest the diagnosis, and in particular with no evidence of intimal disruption[18].

**Table 1:** Angiographic and IVUS features of coronary artery dissection.

	Angiography	IVUS
**Dissection with intimal tear**	radiolucent intimal flap delayed contrast clearance from false lumen	confirmation of true and false lumina identification of intimal tear length and morphology
**Dissection without intimal tear (intramural hematoma)**	narrowed vessel smooth stenosis	confirmation of intramural hematoma length and morphology

Several factors may influence the choice of management strategy in SCAD, including the site of the dissection, the number of vessels involved, distal coronary blood flow, the hemodynamic status of the patient and the availability of coronary intervention. In asymptomatic, stable patients with limited dissections, a trial of medical therapy is recommended, involving beta-blockade to reduce vascular shear forces and anti-platelet agents to reduce thrombus formation. Medical therapy has been reported to result in favorable long-term outcomes, even when multiple vessels are involved [7], and complete angiographic resolution is not uncommon [6]. The use of fibrinolytic agents to dissolve occlusive thrombus within the true lumen, or to lyse compressive clot within the false lumen, remains controversial. The results are unpredictable and intuitively, the use of thrombolysis may worsen ischemia by increasing intramural haemorrhage and propagating the dissection, as in aortic dissection. As in two of our cases, SCAD has been reported as presenting with deterioration after thrombolysis [19].

Therefore, in the setting of SCAD, thrombolysis should be avoided. Instead, PCI is the treatment of choice in patients with single-vessel SCAD with ongoing signs of ischemia. Stenting is effective in pinning back the dissection flap and buttressing against further luminal impingement by intramural hematoma. Although no studies have conclusively demonstrated protection against future dissection, the presence of scaffolding afforded by stent insertion may mitigate against local recurrence. An unresolved issue is whether the whole dissection should be covered or just the inlet, if an intimal tear is identified. Furthermore, it remains unclear whether drug-eluting stents (DES) carry any specific advantage over bare metal stents in the management of SCAD. The use of DES would theoretically minimize the risk of in-stent restenosis in a young patient, though this risk may be low if there is no underlying atherosclerotic disease and one cannot predict the impact of the eluted drug if there is an underlying collagen defect. Any meaningful comparison of bare metal stents and DES in SCAD is not possible currently, and may remain so in view of the rarity of the condition.

A potential complication of stenting includes the extrusion of intramural thrombus up- or downstream of the stent, causing propagation of the dissection. In one study, stent implantation caused propagation of dissection in two out of five cases when IVUS was not used to make the initial diagnosis. [18]. Another concern is the potential for wiring the false lumen, leading to inappropriate stent deployment. This risk may be minimised by injection of contrast through a catheter or over-the-wire balloon passed into the distal vessel before stent deployment. An alternative approach is the use of IVUS guidance, to identify the false lumen and ensure correct wire placement within the true lumen. IVUS is also of great benefit in guiding stent sizing, and following stent deployment, in confirming optimal stent expansion and obliteration of the false lumen.

CABG has been used successfully in multivessel dissections, SCAD involving the LMS and when PCI has failed. Although there are, in the literature, many successful reports of such a strategy, unsuccessful surgery is unlikely to be reported. The major difficulty with this approach is the risk that the true lumen cannot be clearly identified, leading to grafting of the false lumen, with subsequent irreversible myocardial damage or death.

In our series, all four cases of SCAD occurred in young or middle-aged females, three involving the LAD and one, the RCA. In two cases, the diagnosis of SCAD was suspected following clinical deterioration after administration of thrombolytic agents. Two cases were managed conservatively, with symptoms and angiographic abnormalities resolving with medical treatment; the remaining cases underwent successful PCI. IVUS was used in to confirm the diagnosis, to guide PCI and to confirm sealing of the dissection and adequate stent expansion.

## Conclusion

SCAD is associated with significant morbidity and mortality. Its pathophysiology is poorly understood but the existence of diverse histological features and aetiological factors suggests that SCAD may not be a single pathological entity. A high index of suspicion is imperative when faced with acute coronary syndromes in peripartum females or in young patients without conventional risk factors. The diagnosis cannot be excluded by coronary angiography, and in cases where doubt remains, IVUS should be utilised to interrogate luminal narrowings, especially in the vessel supplying the territory of interest.

For single-vessel dissections where antegrade flow is preserved and symptoms of ischemia are absent, conservative medical management is recommended. However, thrombolysis may worsen ischemia by increasing intramural hemorrhage and therefore should be avoided. For single-vessel dissections with ongoing ischemia, PCI is the management of choice. For dissections involving multiple vessels or the left main stem coronary artery bypass surgery may be considered.

IVUS should be used in all PCI for suspected SCAD, to:

1. Identify the true lumen

2. Identify intimal tears or intramural hematoma

3. Allow placement of the angioplasty guide wire within the true lumen

4. Guide stent sizing, length and optimal deployment

5. Ensure obliteration of the false lumen and flow within it, or adequate compression of intramural hematoma.

## Competing interests

The authors declare that they have no competing interests.

## Authors' contributions

JRA had the initial idea for the manuscript and wrote the initial draft. NEJW, TDK, WVG and APB revised the article critically. APB managed the cases as interventional cardiologist, and is guarantor. All authors read and approved the final manuscript.

## Consent

Written informed consent was obtained from the patients for publication of this case report and any accompanying images. Copies of the written consent are available for review by the Editor-in-Chief of this journal.

## Funding

APB is partially funded by the Oxford Biomedical Research Centre.

## Supplementary Material

Additional file 1 Coronary angiogram (case 1). RAO caudal projection showing LAD dissection. Click here for file

Additional file 2 Coronary angiogram (case 1). RAO caudal view of the LAD following PCI. Click here for file

Additional file 3 Coronary angiogram (case 4). LAO view showing tubular stenosis in RCA. Click here for file

Additional file 4 IVUS recording (case 4). IVUS examination showing intramural hematoma. Click here for file

Additional file 5 Coronary angiogram (case 4). LAO view showing RCA at follow up. Click here for file
